# Quality of Obstetric Referral Services in India's JSY Cash Transfer Programme for Institutional Births: A Study from Madhya Pradesh Province

**DOI:** 10.1371/journal.pone.0096773

**Published:** 2014-05-08

**Authors:** Sarika Chaturvedi, Bharat Randive, Vishal Diwan, Ayesha De Costa

**Affiliations:** 1 Department of Public Health and Environment, R D Gardi Medical College, Ujjain, India; 2 Department of Public Health Sciences, Karolinska Institutet, Stockholm, Sweden; 3 Department of Public Health and Clinical Medicine, Umea University, Umea, Sweden; 4 International Centre for Health Research, R D Gardi Medical College, Ujjain, India; Iran University of Medical Sciences, Iran (Islamic Republic Of)

## Abstract

**Background:**

India launched JSY cash transfer programme to increase access to emergency obstetric and neonatal care (EmONC) by incentivising in-facility births. This increased in-facility births from 30%in 2005 to 73% in 2012 however, decline in maternal mortality follows a secular trend. Dysfunctional referral services can contribute to poor programme impact on outcomes. We hence describe inter- facility referrals and study quality of referral services in JSY.

**Methods and Results:**

Women accessing intra natal care (n = 1182) at facilities (reporting >10 deliveries/month, n = 96) were interviewed in a 5 day cross sectional survey in 3 districts of Madhya Pradesh province. A nested matched case control study (n = 68 pairs) was performed to study association between maternal referral and adverse birth outcomes. There were 111 (9.4%) in referrals and 69 (5.8%) out referrals. Secondary level facilities sent most referrals and 40% were for conditions expected to be treated at this level. There were 36 adverse birth outcomes (intra partum and in-facility deaths). After matching for type of complication and place of delivery, conditional logistic regression model showed maternal referral at term delivery was associated with higher odds of adverse birth outcomes (OR- 2.6, 95% CI: 1.0–6.6 p = 0.04). Maternal death record review (April 10–March 12) was conducted at the CEmOC facility in one district. Spatial analysis of transfer time from sending to the receiving CEmOC facility among in-facility maternal deaths was conducted in ArcGIS10 applying two hours (equated to 100 Km) as desired transfer time. There were 124 maternal deaths, 55 of which were among mothers referred in. Buffer analysis revealed 98% mothers were referred from <2 hours. Median time between arrival and death was 6.75 hours.

**Conclusions:**

High odds of adverse birth outcomes associated with maternal referral and high maternal deaths despite spatial access to referral care indicate poor quality of referral services.

## Introduction

Maternal mortality is considered to be the greatest health inequity of the 21^st^century [1]. It continues to be a problem largely for poor women in low and middle income countries (LMICs).Five obstetric complications cause over two thirds of maternal deaths [2].While most fatal complications cannot be prevented or predicted, they can be effectively treated if women have access to good quality emergency obstetric care (EmOC). Given that most maternal deaths occur during labour, delivery and the first 24 hours post partum, an effective intra partum care strategy including EmOC services has been identified as a priority to reduce maternal deaths[3] [4].

India, which contributes to a fifth of all maternal deaths, adopted an intra-partum care strategy, under its Janani Suraskha Yojana (JSY) program. The JSY, launched in 2005, is a cash transfer paid to women when they give birth in a health facility. The objective of the JSY has been to reduce maternal and neonatal mortality by promoting in-facility delivery. The criteria for women to qualify as beneficiaries, as well as the size of the cash transfer, vary between provinces of the Indian Union [5]. The program has thus far had 54 million beneficiaries. National surveys [6] [7] have documented a steep increase in institutional delivery proportions since the JSY began- from 30% in 2005 to 73% in 2012 [8].Studies published to date have failed to detect an effect of the JSY on reduction in maternal [9] [10] and neonatal mortality [11].

Effective referral services are central to a program like the JSY, which aims to provide EmOC care to save lives. It is known that reductions in maternal mortality and morbidity are not possible without an effective referral system for obstetric complications [12].Obstetric complications are unpredictable and offer a short time window to start medical care, failing which they can be fatal. The capacity of different tiers of public sector health facilities (that are all also JSY institutions) to function as EmONC facilities is varied, with some being competent Comprehensive emergency obstetric and newborn care (CEmONC)facilities, while others function at less than Basic EmONC (BEmONC) levels. Given this variation, it is all the more important that an effective referral system is in place to facilitate effective first line management at the first facility a mother attends, and efficient transfer to more comprehensive care facilities when complications may necessitate this. A dysfunctional referral system can contribute to a poor program impact on maternal and neonatal mortality outcomes.

Despite the criticality of obstetric referral services for effective EmONC and reductions in maternal and neonatal mortality, no studies have examined the functioning of these, especially in the context of the JSY program in India. Questions that remain unanswered are about the processes and characteristics of referral like proportion of mothers participating in the program that are referred, indications for which they are referred, facility levels from (and to) which referrals are made, transport used for referral, outcomes of delivery in referred mothers and their newborns. Importantly the quality of referral services in the program also needs study. Thus the objectives of this study were (1) to describe the inter-facility obstetric referrals in the JSY cash transfer programme in terms of (a) the proportion of mothers referred (b) referral indications (c) referral patterns and (d) delivery outcomes, and (2) to study the quality of referral services in the JSY by examining (a) association between maternal referral and birth outcomes and (b) spatial access to CEmOC among mothers who were referred to and subsequently died at a CEmOC facility.

Large investments are underway in a number of LMICs to strengthen their capacities to provide EmOC and achieve MDGs 4 and 5, however research into the functioning of the referral services for EmOC in these areas is limited [13]. This study of referral services for EmOC in the JSY in India has implications for the JSY program as well as for other low middle income settings working to strengthen EmOC to achieve their MDGs.

### Study Framework

**Figure 1:**
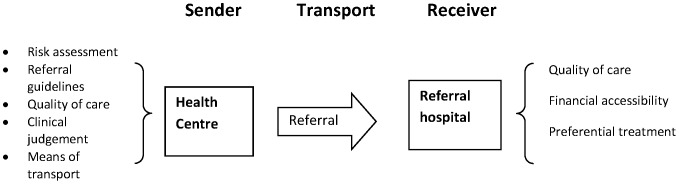
Model of referral chain, adapted from Jahn A and De Brouwer V 2001.

The term referral is often used to indicate an advice given by a health worker to seek care at higher level facility, whether followed or not [14]. In this study, within the context of the JSY program, we used the term referral to mean movement of women seeking intra-natal care from one health facility to another, because of an inability to receive the necessary obstetric care at the first facility attended. We used the referral chain model proposed by Jahn et al[15]. The model conceptualises referrals as composed of three main components- sender, transport and receiver. Although the importance of the family or village level in the referral chain in Jahn's model cannot be denied, we limited this study to referrals between facilities, as this is relevant in the context of the JSY. An adaptation of Jahn's model of the referral chain that focuses on the sending and receiving facilities in the JSY program is shown in Figure 1.

## Methods

### Study site and context

The study was conducted in Madhya Pradesh (MP) province in central India. MP has a population of 72 million, 70% of which is rural [16]. It has relatively poor health indicators compared to the rest of the country (Table 1). The public sector, through which the JSY is implemented, is the dominant provider of obstetric services in the province. The private sector is relatively small and concentrated in urban areas.

**Table 1:** Profile of study districts.**

Health/Development indicators	District 1	District 2	District 3	Madhya Pradesh	India
Maternal mortality ratio*	415	386	206	277	212
Neonatal mortality rate	46	65	32	43	33
Literacy (%)	69	67	74	74	74
Institutional delivery (%)	58	72	81	76	73
Human development Index	0.5	0.4	0.6	-	
Population(million)	1	1	1.9	72	1000

^*^MMR estimates are regional;

^**^ Most recent figures available at time of district selection.

Source-Annual Health Survey and Census of India 2011, Registrar General of India, Government of India;,Government of India; Human Development Indices of districts of MP 2001.

The province is divided into fifty independent administrative districts. Each district has approximately a million people and a separate district health department that oversees health facilities, health workers and program implementation within its boundaries. Three districts were purposively selected for this study to reflect different levels of maternal mortality, institutional delivery proportions, and heterogeneity in socio demographic profiles and variations in geographical parts of the province. (Table 1).

The JSY: In MP, the JSY provides a cash transfer to all mothers (∼31 USD if rural, 22 USD if urban) conditional on delivering in public health institutions. Delivery care in public institutions is formally free of charge. All public health facilities, regardless of the level of functionality are eligible for the JSY i.e. they provide the cash incentive to mothers delivering there. The program has raised institutional birth proportion in MP from 30% in 2005 to 81% in 2010[17].Thus far, 7 million women have been beneficiaries of the program across the province [18].Apart from the cash incentive to mothers, under the JSY programme; the government also provides a cash incentive to rural female health volunteers called ASHAs (Accredited Social Health Activists) to escort women to institutions for delivery.

The public health system and referrals: The public healthcare delivery system is organised in three tiers comprising Primary Health Centres (PHCs), Community Health Centres (CHCs) at secondary level and tertiary District Hospitals (DHs). The PHCs are expected to provide normal delivery care and referrals when there are complications; the CHCs are expected to provide specialist services, and some of these are designated as First Referral Units (FRUs) providing Caesarean section while the district hospitals are expected to be tertiary care facilities handling complicated cases. Although varying by district size, on average each district has about 40 PHCs, 6–9 CHCs and one DH. While this hierarchy of healthcare facilities exists, patients can choose to access any level of care directly, without moving through the hierarchy. There is no formal preferential treatment for referred cases. Ideally when patients are referred from one facility to another, a referral slip is to be sent with the patient and a duplicate record is maintained at the sending facilities, although this is not always done in practice. There is no reply form or information sent back from the receiving facility to the sending facility on the case referred. The MP provincial government operates a transport system called Janani Express (JE)[19] (started 2006) to transport pregnant mothers to/from or between health facilities. The JE service is formally free of charge.

### Study design

The objectives of this paper were addressed through different studies. The first objective to describe the inter facility referrals was studied using a cross sectional survey of mothers delivering under the JSY program. The quality of referral services is studied in two ways (a) by using a nested matched case control design to examine association between maternal referral and birth outcomes, and (b) a retrospective maternal death record review to examine spatial access to CEmOC.

### Data collection

#### Cross sectional survey

A list of delivery facilities along with the average number of deliveries each facility performed was obtained from the district health office in the three districts. The facilities in three study districts- both public and private that performed 10 or more deliveries a month were invited to participate in a cross sectional survey. At the time of the survey, researchers visiting each facility also enquired with facility personnel about availability of other delivery centres in the vicinity. This way snowballing was used to complete the list of delivery facilities. Trained women research assistants visited each study facility for five consecutive days. All women admitted or referred into a facility for intra natal or early post natal care were interviewed to gather data on socio demographic profile, delivery and referral details. During the same five day period details of obstetric referrals sent out from study facilities were obtained by meeting the nursing staff soon after the referral. The survey was done during February 2012 through to April 2013. Data on reasons for referral were sought from the referral slips and by meting nursing staff at facilities while data on other variables were obtained from mothers interviewed. The description of inter-facility referrals and the nested case control study draw on this survey data.

#### Record review

Data for our spatial analysis was sourced from maternal death records maintained at the only Comprehensive Emergency Obstetric Care (CEmOC) facility in district 1. This facility is the only one that provides services for obstetric complications including Caesarean section and blood transfusion. Maternal death records are required to be maintained at all health facilities in a prescribed format by the government. These records (Apr 2010–Mar2012) were studied at the CEmOC facility to extract data regarding deaths among those mothers referred into this hospital for obstetric care and subsequently died there. In particular data on sending facility locations, delivery details, cause of death, and times of arrival and death were extracted. The two main variables of interest were (a) spatial access measured by travel time between the sending facility and the receiving CEmOC facility and (b) time between the mother's arrival at the CEmOC facility and death. All sending and the receiving facility were geo-positioned onto a digitised map of the district. The distance between the sending and receiving facilities was converted into travel time assuming the average speed of a van in the study area is 50 km/hour. This average for van speed was based on information provided by key informants including nursing staff and ambulance drivers in the study district. Travel time between sending and the receiving facility (the district hospital) was categorised into <1 hour, between 1and 2 hours and >2 hours. Time between arrival and death was derived from the medical record entries.

#### Ethics statement

All the study participants in the survey provided written informed consent to participate. Consent was administered in the local language. The study was approved by the Institutional Ethics Committee of the R D Gardi Medical College, MP, India.

### Analysis

To describe inter facility referrals, we determined the proportion of mothers referred for intra partum care, among those who accessed the study facilities for delivery care during the 5 study days. Referral pathways of mothers who were referred were traced and analysed by facility levels to produce referral patterns. Descriptive statistics were used to analyse data on time spent at facilities in the referral chain, referral indications, distance travelled, transport used and delivery outcomes.

In the matched case control design, cases were women who delivered at term but had adverse birth outcome defined as intra partum foetal death or in-facility neonatal death within 48 hours post partum. Controls were women who delivered at term and had live neonates at 48 hours post partum matched individually to cases for the type of obstetric complication and place of delivery. The exposure of interest was maternal referral. Types of complications matched included (1) haemorrhage (2) pre eclampsia or eclampsia (3) obstructed labour (4) infection and (5) indirect causes (anaemia). The second matching criterion was place of delivery. This was chosen to limit differences in case management and quality of care received. Each case was matched to two controls. Odds ratio and 95% confidence intervals were calculated using conditional logistic regression to study association of referral with birth outcomes controlling for other potential confounders.

A spatial analysis of transfer time from sending to the receiving CEmOC facility among in-facility maternal deaths was conducted. The CEmOC facility and all sending facilities from where the deceased mothers were referred to the CEmOC facility in District 1 were plotted on the map. Buffers of a radius of 50 km (equated to one hour travel time) and 100 km (equated to two hours travel time) were constructed around the CEmOC facility. We used the two hour travel time for categorisation since a travel time of two hours to a CEmOC facility is considered a standard for adequate spatial access to EmOC[20] [21]. Hence deaths among mothers who were referred to CEmOC facility from a facility located within the 2 hour buffer indicated poor quality referral services, either at the sender or receiver levels or at both these levels in the referral chain. Time spent at the CEmOC facility before death was used to assess care at the receiver level facility.

Quantitative data were analysed in STATA. Spatial analysis was conducted using ArcGIS 10.

## Results

### Objective 1: To describe inter facility referrals in the JSY

#### Study facilities and mothers

The facility survey yielded a 97% response rate resulting in 96 facilities included in the study. The study facilities by levels of care are presented in Table 2. All the private facilities surveyed provided Caesarean section and have been categorised separately as they are not JSY program facilities. All the three district hospitals provided Caesarean section, while none of the CHCs (secondary level) except one in District 3 did so. For all primary and secondary level facilities, the respective District Hospitals were the referral centres of first choice equipped with blood and obstetric surgery facilities. The average distance to these higher centres was 47 km (IQR 25–63 km). All facilities had 24 hour seven days a week access to free Janani Express van for referral transportation. The distribution of mothers who accessed intra-partum care at these facilities during the 5 day study period is presented in Table 2.

**Table 2:** Study facilities by level and distribution of mothers who accessed intra-partum care at these levels.

Facility levels/type	No of facilities (%)	No of mothers accessed care (%)
Primary	47 (49)	203 (17)
Secondary	24 (25)	493 (42)
Tertiary	3 (3)	362 (31)
Private	22 (23)	124 (10)
Total	96 (100)	1182 (100)

**Figure 2:**
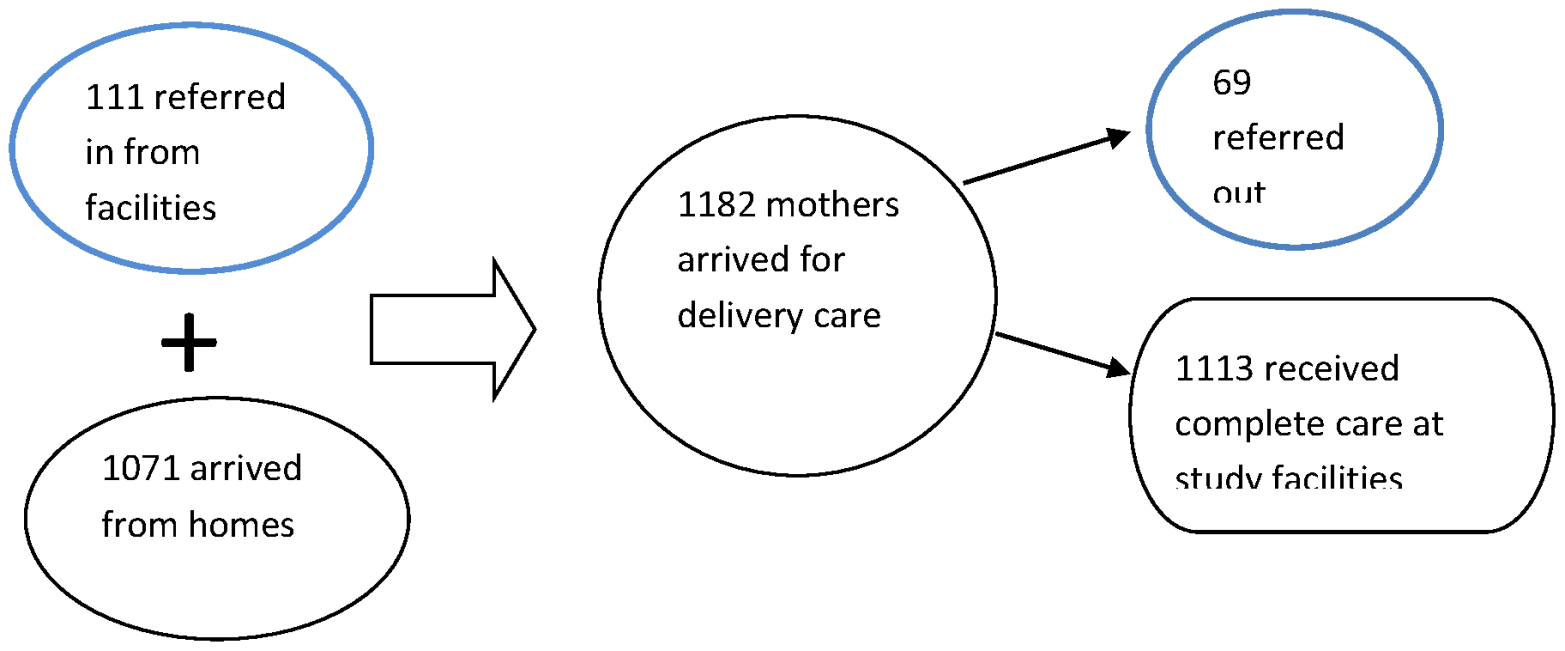
Mothers who reached study facilities for intra-partum care by referral status.

A total of 1182 mothers arrived at the 96 study facilities for delivery care during the 5 day survey period. Of these, 1071 came directly from home while 111(9.4%) came on referral from another facility (Figure 2). Of the 1182 mothers who arrived at the study facilities, 69 (5.8%) mothers were referred out for higher care while 1113 were admitted and received delivery care in study facilities. All the 111 mothers who arrived on referral into study facilities completed their care there, none were referred further on.

In keeping with our objective of studying referrals, we included 180 mothers (111 mothers who were referred into the study facilities and the 69 who were referred out from the study facilities) in this part of study. Of the referred in mothers, 60% belonged to families living below the poverty line; 35% had a caesarean section delivery. Only 4% were grand mutiparas.

#### Referral indications

For the 180 referred mothers, prolonged labour (obstruction/malposition) was the most common reason (39%) for referral followed by leaking (13%), haemorrhage (8%) and pre/eclampsia (7%). Importantly, facility dysfunctionality that included non availability of staff, or power and water shortages at sending facilities was a reason for referral in 7% cases; all such sending facilities were primary or secondary level ones.

Referral indications by level of sending facilities are presented in Table 3. Of the referrals from the CHC level over one third (40%) were for prolonged labour and a fifth were for leaking membranes, both are conditions ideally expected to be managed at the CHC level.

**Table 3:** Reasons for referral by sending facility levels.

Reason for referral	PHC	CHC	DH	Private	Total	%
Prolonged labour	23	42	3	2	70	38.9
Leaking	2	20	0	2	24	13.3
Haemorrhage	6	7	1	0	14	7.8
Pre Eclampsia/Eclampsia	2	7	2	2	13	7.2
Bad Obstetric History	6	3	1	0	10	5.5
Primiparity	4	1	0	1	6	3.3
Anaemia	1	4	0	0	5	2.8
Foetal distress	1	4	0	0	5	2.8
Twins	1	3	0	0	4	2.2
Retained placenta	0	1	0	0	1	0.5
Other reasons(medical)	2	8	1	0	11	6.1
Facility dysfunctionality	8	5	0	0	13	7.2
Not mentioned	2	1	0	1	4	2.2
Total	58	106	8	8	180	100

#### Characteristics of referrals

Of all the referrals, 97% were made prior to delivery. There were 2% referrals that were made directly from the facility entrance without any examination or treatment, 38% after examination, and 60% were admitted into the sending facility before being referred (median duration of admission  = 4.5 hours). A referral slip was given to 72% referred mothers. The referral slip mainly contained patient name, date and the facility to which she was referred. Two thirds (63%) of inter facility transfers used the JE transport. The average inter facility travel time was 1.25 hours.

#### Referral pattern

The referral patterns for referred in and referred out mothers are presented separately below. Of the total 111 referred in mothers, 98 were referred once, 11 were referred twice and two were referred thrice resulting in a total of 126 inter facility referrals among the referred in mothers.

**Figure 3:**
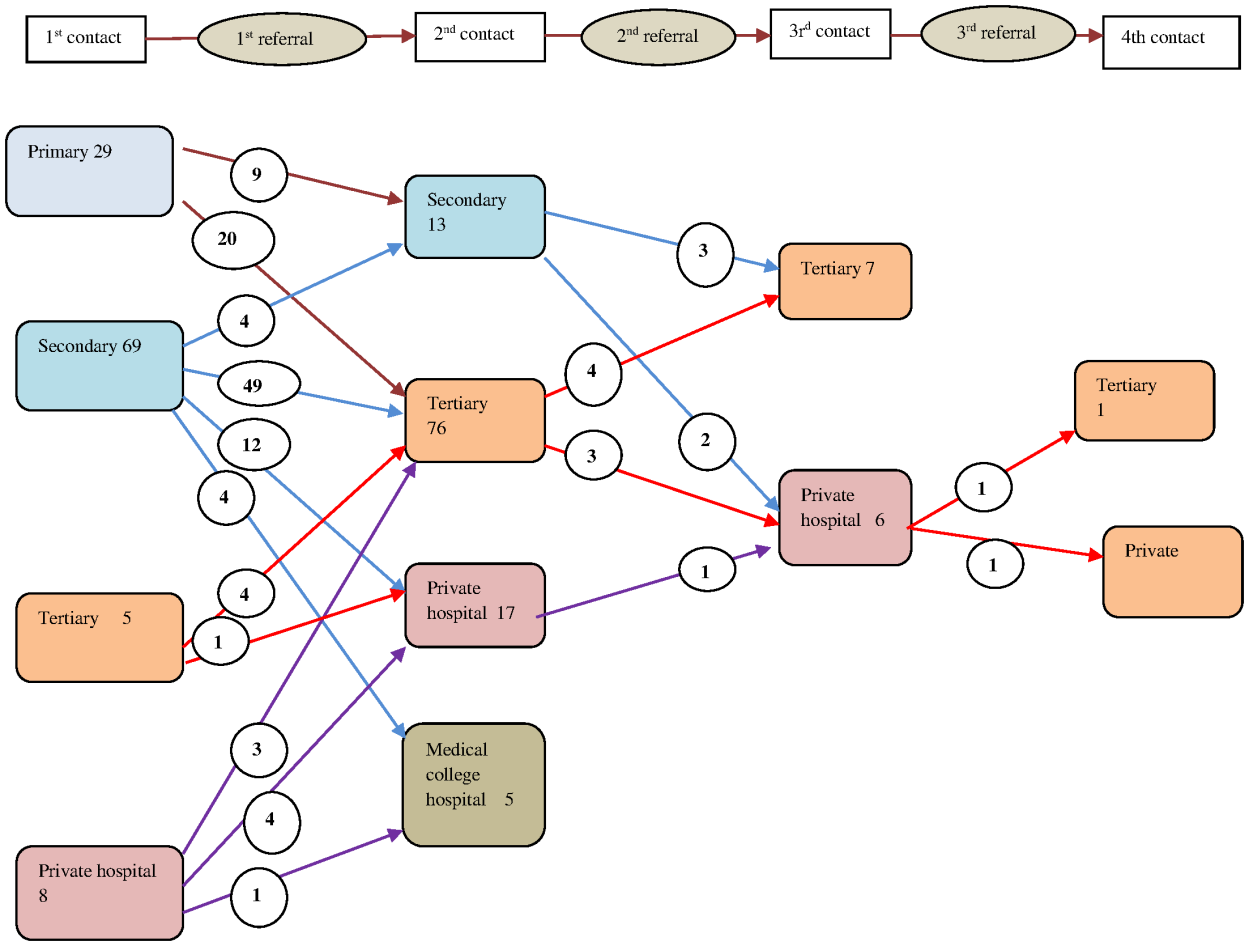
Referrals by facility levels among referred in mothers. (Arrows point to receiving facilities, encircled numbers on arrows indicate numbers of mothers who went to the receiving facility.)

As seen in Figure 3, in the first referral, sending facilities for mothers referred in were secondary level CHCs in 62% of cases and primary levels in 26% of cases. Of mothers referred from both these levels, 70% went to tertiary level receiving facilities in the public sector. This pattern depicted in Figure 3 below shows that the tertiary level facilities receive most referrals, while the secondary level facilties receive very few referrals at all. In all 13 (12%) mothers who had been referred once required a further referral, half of whom went to a private facility, indicating greater involvement of the private sector in higher order referrals.

**Figure 4:**
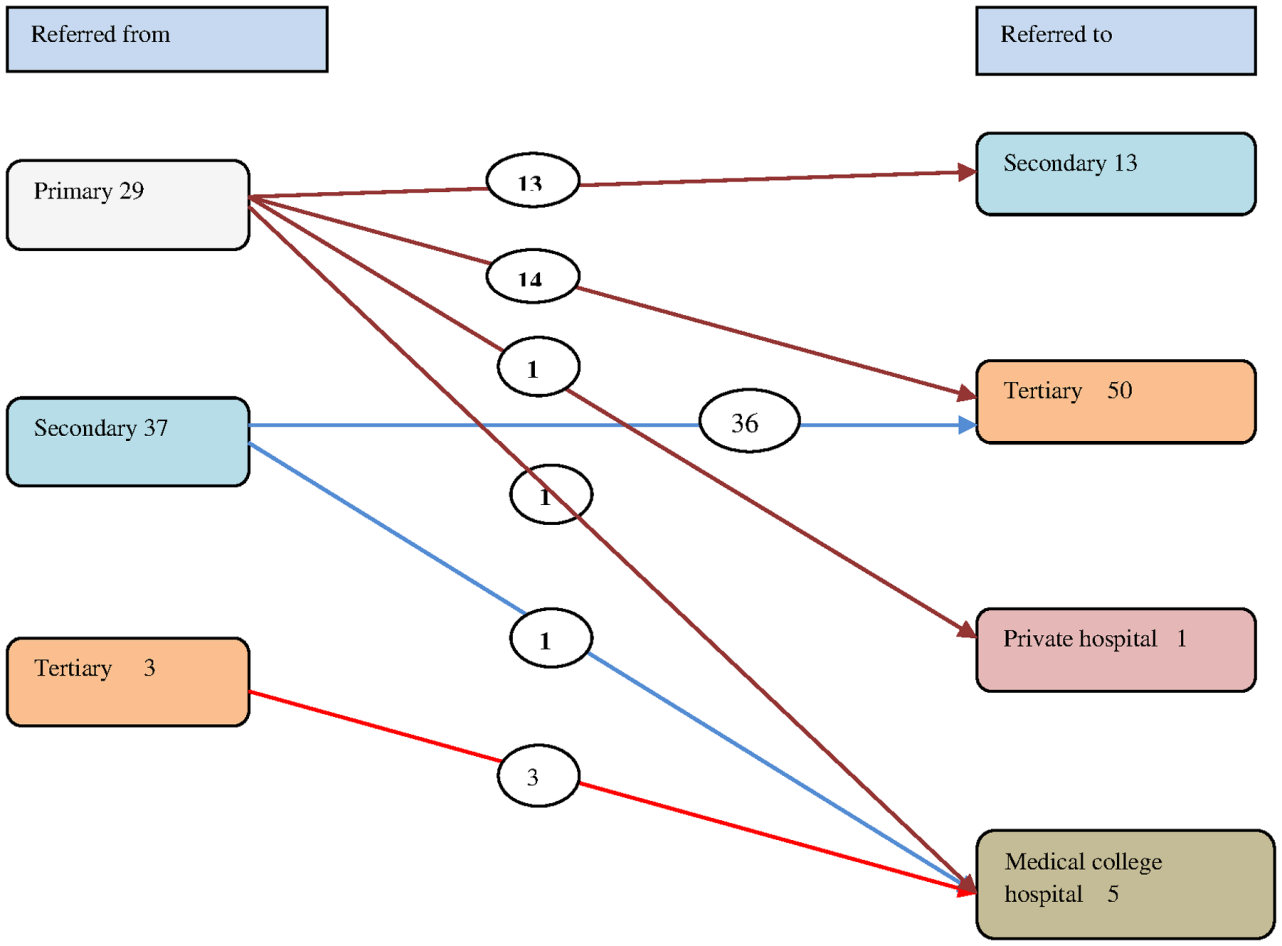
Referrals by facility levels among referred out mothers.

Referral patterns for the 69 mothers who were referred out reveal the same pattern as above –secondary level facilities (CHCs) were the common senders and tertiary level facilities (DHs) were the common intended receivers of referrals. About half the referrals from primary level were sent directly to tertiary level. Referrals were rarely sent from public to private facilities, while few referrals were sent to the medical college. The referral patterns of these 69 inter facility referrals are shown in Figure 4.

#### Delivery outcomes

Outcomes of delivery among mothers referred out during the study period are not known, we present here outcomes for all mothers admitted at study facilities (n = 1113). There were no maternal deaths while 36 adverse birth outcomes (intra partum foetal deaths and early in-facility neonatal deaths) occurred including two in preterm deliveries. The mothers whose infants had adverse birth outcomes had spent a median time of 2 hours at the sending facility.

### Objective 2: To study quality of referral services in the JSY

#### Association between maternal referral and birth outcomes

The median age of cases (n = 34) was 22 years and that of controls (n = 68) was 23 years. When compared to controls, more cases belonged to families living below the poverty line (65% vs 41%) and received a referral (50% vs 25%p<0.05) An equal proportion(32%) in the two groups were illiterate and 50% of the cases and 44% of controls were primiparous.(Table 4).

**Table 4:** Descriptive data for cases and controls.

Characteristic	Cases (n = = 34)	Controls (n = 68)
Age in years, median (range)	22 (18–35)	23 (18–40)
Below poverty line*	65%	41%
Illiterate	32%	32%
Referred*	50%	25%
Primiparous	50%	44%
Caesarean section delivery	15%	28%
ANC visits <2	38%	25%
Delivery care by doctor	21%	30%

*Difference between cases and controls significant P<0.05.

In the matched design, odds of adverse birth outcomes were higher in mothers referred for obstetric care (OR = 2.3 (95% CI: 1.1–5) than those not referred. When potential confounding from age, education, parity, poverty, antenatal care, and provider at delivery was controlled in the conditional logistic regression model, the odds of adverse birth outcomes among women who were referred remained significantly higher(OR = 2.6, 95% CI: 1.0–6.6 p = 0.04)(Table 5).

**Table 5:** Output of conditional logistic regression for association between maternal referral and adverse birth outcomes.

Variable	Odds ratio (95% CI)	P value
Maternal referral	2.61 (1.08; 6.6)	0.04
Age	1.83 (0.60, 5.51)	0.28
Education	1.2 (0.43–3.43)	0.70
Below poverty line	2.06 (0.80–5.31)	0.13
Parity	0.63 (0.19–2.02)	0.43
ANC visit	1.19 (0.57, 2.48)	0.63
Provider	0.30 (0.07, 1.25)	0.10

In the matched design when examined separately, odds for intra partum foetal deaths were higher (OR = 3.1, 95% CI: 1.3–8.7) than those for early neonatal death (OR = 1.3, 95% CI: 0.3–4.9).When association of referral with intra partum foetal deaths was examined in the conditional logistic regression model, maternal referral had four times higher odds of intra partum foetal deaths (OR = 4, 95% CI 1.2–13.8) and the type of provider showed a protective effect – such deaths were less likely in deliveries conducted by doctors than those conducted by nursing personnel or other unqualified staff (OR = 0.15).

#### Spatial access to CEmOC among mothers who were referred to and subsequently died at CEmOC facility

**Figure 5:**
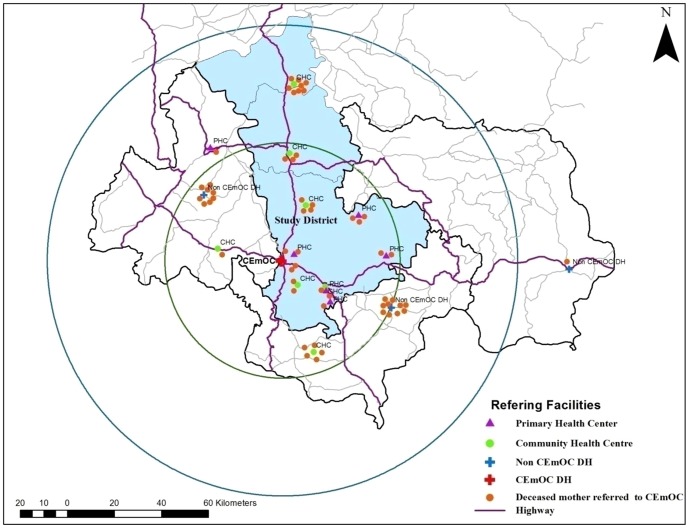
Buffer analysis of distance between CEmOC facility and facilities that referred the 55 mothers who died at CEmOC facility. (Circles in the figure are buffers with radius of 50 Km and 100 Km around the CEmOC facility.)

The single CEmOC facility in District 1 conducted 10474 deliveries during the two study years (April 2010 to March 2012). There were 124 maternal deaths in the facility during this period. Of the 124 maternal deaths, 55 (44%) were among mothers who were referred to the CEmOC facility from another sending facility. These 55 deaths and were included in this part of the study, excluding the rest. Most referrals were from CHCs (Figure 5). Referrals were also received from other tertiary level facilities in neighbouring districts.

Table 6 presents the age, parity, and causes of deaths for the 55 referred mothers. The buffer analysis of travel time between sending facilities and the CEmOC facility (receiver) is shown in Figure 5. It reveals that for 54 maternal deaths (98%) the sending facilties were located within a two hour buffer from CEmOC facility indicating good geographic access. The median time between arrival of mothers at the CEmOC facility and death was 6.75 hours (IQR-2.1–32 hours).

**Table 6:** Details of in-facility maternal deaths who were referred to CEmOC facility (n = 55).

Details of mothers who were referred to CEmOC facility and died (n = 55)		
Median age		25years (Range 19–35)
Primiparous		26 (47%)
Death	Before delivery	16 (29%)
	Post abortion	2 (4%)
	After Vaginal delivery	27 (49%)
	After C section	10 (18%)
Cause of death	Haemorrhage/Anaemia	25 (45%)
	Hypertensive disorders	15 (27%)
	Sepsis	6 (11%)
	Obstructed labour	6 (11%)
	Indirect causes	3 (4%)
Mothers who were referred from facilities <2 hr distance from CEmOC facility		54 (98%)
Mothers who were referred from facilities <1 hr distance from CEmOC facility		45(82%)
Median time between arrival and death at CEmOC facility		6.75 Hours (IQR-2.1–32)

## Discussion

Global maternal and neonatal health outcomes could be significantly improved if ways of overcoming barriers to wide use of effective interventions including EmONC are in place, especially in low income settings. Conditional cash transfers are an increasingly popular mechanism to address the demand side barriers to utilisation of services. The JSY cash transfer program in India has sharply increased the utilisation of institutional care for delivery and thus is assumed to have increased access to EmONC. In order to ensure access to EmONC, a well functioning referral system is a critical more so when demand has been inflated through financial incentives and different levels of facilities in the program are known to have different functional capacities with regard to EmONC services. By systematically studying the referral services under the JSY, we identify the current gaps that need to be addressed to ensure access to effective EmONC under the JSY. This also provides lessons for policy makers interested in adopting CCTs, especially for attaining MDGs 4 and 5.

### Deaths despite access to referral facilities indicate poor quality referral services

The high numbers of maternal deaths inspite of access to a functioning CEmOC facility under the JSY scheme indicate ineffective referral services at one or more of the levels in the referral chain. This could result from multiple causes as shown in Jahn et al's model for referral services in Figure 1, however issues at receiving facility (and possibly) the sending facilities in our study appear more important than those related to transport as most mothers had arrived from facilities within the optimal two hour travel time. Availability of free transport services and the reasonably good condition of major roads in the study district (as found by our research team that travelled on these roads for the survey of health facilities) make problems with transport less likely in this context. Delay in timely recognition of the condition at the sending facility could be an important reason contributing to ineffective referral services and ultimately morbidity/mortality in the study area. In a separate study (unpublished), we found that nurse birth attendants at facilities included in the present study had poor competence at diagnosing and initiating first line management of obstetric complications. Such compromise in role of sending facilities in the referral system could lead to deterioration of the condition and negatively influence outcomes. While the purpose of referrals is to increase likelihood of better birth outcomes, the result from our matched case control study show referral was associated with worse birth outcomes even after controlling for complications and other confounders. Although we could not account for the severity of the complication and are limited by of sample size and diagnostic accuracy, our findings indicate that use of referral services did not ensure access to appropriate life saving care. Our findings are in concordance with a recent study at a tertiary care hospital in urban India that reports lack of care before referral is a predictor of neonatal mortality [22]. A recent multi-country cohort study which included India reports most still borns were fresh (not macerated), born at term deliveries and had normal birth weight (>2500 gm), indicating compromised intra partum care and high proportion of potentially preventable still births in LMICs[23]. Also our finding of a protective effect of deliveries conducted by doctors compared to other staff suggests need for further exploration into competence of other staff at providing intra-partum care, and could be a possible reason for the bypassing of lower and mid level facilities. Most maternal deaths among referred mothers who died at CEmOC facility in our study were due to haemorrhage. Mothers with haemorrhage face a risk of death within 2 hours after the onset of the complication in the absence of medical treatment [24] [25].Since these mothers were referred from a facility that should be able to provide first line management and died at a facility equipped to treat haemorrhage, it is possible that these mothers did not receive basic essential care like intra venous oxytocin and intra venous fluids at the sending facility prior to referral. Besides lack of care at the sending facilities, there are possibly problems in appropriate management of haemorrhage at the CEmOC facility, given that mothers spent a median of 6.75 hrs there before death. This long interval between arrival of referred mothers and their death at the CEmOC facility is indicative of critical delays in providing adequate emergency care, leading to deaths.

Although we found obstructed labour to be the commonest indication for referral, haemorrhage and eclampsia were the leading causes of deaths among referred mothers indicating that inefficiencies in management are more likely in these cases. Increased risk of in-facility mortality in women with haemorrhage and prolonged labour, and if they are referred from another facility has been reported from a cluster randomised trial in Mali [26]. It is important to note that time to death after onset of complication in absence of appropriate medical care is relatively shorter in haemorrhage - 2 hours than in obstructed labour (12 hours) [20]. Our findings hence indicate inability at providing a rapid response in life threatening conditions, especially haemorrhage, and compromise in the quality of care at the receiving CEmOC facility.

Our finding is in agreement with conclusions of a systematic review of ‘third delay’ to explore reasons for maternal deaths despite institutional care by Knight et al that highlights how a focus on patient side delays can conceal that many health facilities in low income settings are yet unable to effectively treat serious obstetric complications [27]. Findings similar to ours are reported from other high maternal mortality regions- a maternal death inquiry in African region[28] and a study in Ghana[29] reported that a large part of avoidable maternal deaths were due to problems in managing emergencies at hospitals. Dogba et al in a study of maternal referral system in Kayes, Mali show that that maternal-newborn survival in the referral system is influenced by combined effects of point of care, the skill configuration of CHC personnel and distance travelled [30].

Improvement in performance at referral facilities under the JSY is essential for access to EmONC to be effective. Besides death reviews that are recently being emphasised under the JSY in India, routine measures like number of deliveries with adverse outcomes, number of such events per delivery and severity of outcomes could be useful [31] as also near miss audits and hospital standardised mortality rates[32].

### Dysfunctional secondary level facilities and overcrowding at tertiary level facilities

The referral patterns and indications in our study show secondary level facilities in the JSY scheme merely serve as sending facilities. They received very few referrals and were unable to handle obstetric complications- 40% referrals from CHCs were for obstructed labour. The practice of direct referrals sent from primary to tertiary referral level facilities and not to mid level facilities as found by us, was also reported over decade ago by a study from India [33], and more recently from Tanzania [34]. Perceived poor quality of care at bypassed facilities was an important reason for the bypassing behaviour. In the present study context, where most mid (secondary) level facilities lack the ability to provide even basic EmOC, patient interests are best served by direct referrals made to tertiary level facilities. This puts an additional burden on the higher level facilities compromising their quality of care. Preferential treatment to referred cases is mentioned as one of the influencing factors at receiving level in Jahn's model. A system of triage at receiving facilties to identify mothers requiring emergency care and those requiring additional monitoring would be beneficial. A study from Rajasthan, India reports use of colour coded cards sent with patients referred for EmOC helped in seizing immediate attention of staff at referral facilities [35]. Another noteworthy example in this regard comes from the UK where hospitals had ‘functional splits’- complicated cases were treated in separate wards by specially trained senior providers while others cared for non-complicated births [36], as also in a hospital in Nepal[37].

### Provider practices regarding referral

Although we found referral slips were provided to most referred mothers, there was no mention of adequate details of treatment provided and progress of the labour. Our findings indicate lack of adequate communication between sending and receiving facilities.

Authors of a recent maternal death enquiry in a district of Madhya Pradesh report that staff refrain from intervening in complicated cases and attempt to evade responsibility by referring out mothers with complications. It is likely that staff fear being blamed by patients' families and questioned by higher authorities in the event of adverse outcomes at their facility premises. [38]. Such practices place women at further risk of death in multiple transits as has been reported by George et al from a south Indian province [39].

The referrals sent from district hospitals in our study were for conditions that ordinarily ought to be managed there. Though referrals sent out from district hospitals were few in number, this is concerning as it indicates failure to provide required care at the highest centres for the JSY program in the district. Such patients are usually sent to medical colleges for critical care; which then does not necessarily remain free of cost to users as intended by the JSY program. In our setting, referrals from district hospitals possibly happen when either an obstetrician or an anaesthetist is not available because precarious human resources do not allow adequate cover when obstetricians are deputed to conduct sterilisations in various facilities or are on leave.

### Weak monitoring and accountability measures

Our findings indicate that there are inefficiencies in the referral services studied and that these are complex, multi factorial and demand a systemic approach. The inefficiencies this study finds could possibly stem from a varying mix of incompetence of providers at sending facilities at recognition and stabilisation of obstetric emergencies, lack of motivation of providers to perform to the best of their abilities contributing to phase III delays (delays in receiving appropriate care on reaching a health facility [40]), a lack of monitoring of referral services and poor accountability of the system to the users. Although all these elements have not been explored in our study, overall it shows weaknesses in the referral system in the JSY and calls for measures to improve the functioning of these critical elements. It is important to emphasise what Murray et al. [41] include in their requisites for successful referral system- supervision and accountability of providers performance and the capacity to monitor effectiveness. The need to improve monitoring of referral services in developing contexts is documented [13].Suggested methods of monitoring referrals include use of specific comparable indicators for referral service, conducting audits [42] [43] and GIS modelling combined with population data[44]. Our findings highlight the need to institute effective data recording on referrals and appropriate monitoring mechanisms to support improvement in the referral process while reemphasising the criticality of effective referral services for EmONC in the context of a CCT programme for institutional delivery care.

### Methodological discussion and limitations

First, the delivery outcomes of mothers referred out from study facilities are not known. However the pattern is unlikely to be very different from those of mothers referred into study facilities and included in our study. Secondly, it could be argued that findings could vary at different times and in different settings. Although generalizability of our findings is limited, we believe that the key action areas we draw up are likely to be similar in other low income settings. For the spatial analysis, our assumption of van speed of 50 km/hour and hence 100 km distance being the equivalent to 2 hours of travel time, was arrived at from the triangulation of information from van drivers, nurses and our own experience of using these roads. Even if one was to assume this speed to be less or more by 10 km/hour, our results remain unchanged. In this part of our study on in -facility maternal deaths among users of referral services; it is not known if mothers arrived early on onset of complication or not and what modes of transport were used. We would argue though that once the mothers reach a health facility, the responsibility for care rests with the services including referral services and not with the users. This is in line with Knight et al who caution against shifting the blame to users in situations when facilities are dysfunctional. Fourth, the staff at participating facilities could have reported reasons for referral differently leaving a potential for misclassification of referral indications in our descriptive results, although this non-differential does not affect results from our matched case control design.

Birth weight and congenital anomalies could be possible confounders not considered in our case control study; however we were limited by inability to obtain reliable data on these variables and suggest interpretation of our findings in this consideration. Also our study has wide CIs because of small numbers and our focus being inter facility referrals, we have not studied the family/community level in the referral chain. Our sample had 34 cases and 68 controls. Given that referrals were 50% in cases and 25% in controls, our study had a power of 47% at alpha = 0.05. Therefore our results should be interpreted with this caution.

## Conclusions

To the best of our knowledge, this is the first study of referral services in the context of the JSY in India. We report on important aspects that have thus far remained under researched. This paper demonstrates how a simple GIS method - buffer analysis of transfer time among deaths in obstetric referrals can be used as a tool to assess quality of referral services.

The JSY cash transfer intervention is currently implemented through facilities that are not backed by good quality referral services. Hence these facilities fail to provide the life saving care essential for achievement of the program goals to reduce maternal and neonatal mortality. Our findings call for attention of policy makers and managers of the JSY program to monitoring effectiveness of referral services. An urgent improvement in referral system by ensuring first line care before referral, care during transfer, appropriate and preferential treatment to referred cases at referral facilities besides strengthening sub optimally performing facilities to function at desired levels is recommended Countries interested in raising demand for services by way of conditional cash transfer schemes should necessarily ensure a strong supply side prior to adopting JSY like schemes.
